# Comparative radiologic characteristics of isotretinoin-induced sacroiliitis and axial spondyloarthritis

**DOI:** 10.1007/s11845-026-04414-y

**Published:** 2026-04-21

**Authors:** Mehmet Gurdal Demirci, Nurcihan Yavuz Savas, Ozlem Kudas, Muhammed Aslan, Toktamis Savas, Fatma Elif Yildirim, Irada Ibramkhalilova, Fatih Albayrak, Orhan Zengin, Bunyamin Kisacik

**Affiliations:** 1 Department of Radiology, Faculty of Medicine, Sanko University, Gaziantep, Turkey; 2 Department of Rheumatology, Istanbul Physical Medicine and Rehabilitation Training and Research Hospital, Istanbul, Turkey; 3 Department of Internal Medicine, Faculty of Medicine, Sanko University, Degirmicem, Gazimuhtar Pasa Bulvarı No:36, 27090 Sehitkamil/Gaziantep, Turkey; 4 Department of Orthopaedics and Traumatology, Faculty of Medicine, Sanko University, Gaziantep, Turkey; 5 Department of Dermatology, Faculty of Medicine, Sanko University, Gaziantep, Turkey; 6 Division of Rheumatology, Department of Internal Medicine, Faculty of Medicine, Sanko University, Gaziantep, Turkey; 7 Division of Rheumatology, Department of Internal Medicine, Faculty of Medicine, Gaziantep University, Gaziantep, Turkey

**Keywords:** Sacroiliitis, Axial spondyloarthritis, Isotretinoin, Acne vulgaris, Inflammation

## Abstract

**Background:**

Sacroiliitis is a major cause of inflammatory low back pain in young adults and a key feature of spondyloarthritis (SpA), but it may also occur with infection, pregnancy, or drug exposure. Isotretinoin, widely used for severe acne, can rarely induce sacroiliitis.

**Aim:**

This study aimed to compare the MRI characteristics of isotretinoin-induced sacroiliitis with those of axial spondyloarthritis (axSpA).

**Methods:**

The study population consisted of 68 patients, comprising 28 with isotretinoin-induced sacroiliitis (median age 24 years; 5 males) and 40 with axSpA (median age 30 years; 26 males). MRI scans were assessed for active inflammatory lesions (bone marrow edema, synovitis, capsulitis, enthesitis) and early structural lesions (erosion, fat metaplasia). SPARCC scoring was performed using the standardized 0–72 system. Ankylosis and sclerosis were not included as it was absent in both groups. Group comparisons were conducted using the Mann–Whitney U test, chi-square test, or Fisher’s exact test.

**Results:**

While isotretinoin-induced sacroiliitis showed a purely inflammatory pattern without structural lesions, patients with axSpA exhibited both active inflammatory and structural lesions. Erosions and fat metaplasia were observed exclusively in axSpA patients (*p* < 0.001). Bilateral and symmetric involvement, as well as higher SPARCC scores, were significantly more frequent in axSpA (*p* < 0.05).

**Conclusion:**

Isotretinoin-induced and axSpA sacroiliitis exhibit distinct MRI characteristics. Erosions and fat metaplasia are specific markers of axSpA, whereas isotretinoin-induced sacroiliitis represents a reversible, non-destructive inflammatory process. The present results may assist clinicians and radiologists in distinguishing between these two conditions in clinical practice.

## Introduction

Sacroiliitis, defined as inflammation of the sacroiliac joints, is a leading cause of inflammatory low back and buttock pain in young adults. Early diagnosis is critical to slow disease progression and prevent irreversible structural damage. Imaging modalities with high sensitivity and specificity are therefore essential for sacroiliac joint assessment. Magnetic resonance imaging (MRI) is regarded as the gold standard, as it enables visualization of both active inflammatory lesions—such as subchondral bone marrow edema—and structural changes, including erosion, sclerosis, fat metaplasia, and ankylosis [1].

Axial spondyloarthritis (axSpA) refers to a group of chronic, systemic inflammatory disorders that primarily affect the axial skeleton, particularly the sacroiliac joints. It is widely recognized as the leading cause of sacroiliitis and is typically characterized by bilateral and symmetric joint involvement, a key diagnostic feature of the disease. The MRI features of axSpA-related sacroiliitis have been standardized by the Assessment of SpondyloArthritis International Society (ASAS) and are widely used in research settings and clinical practice for classification and assessment of inflammatory activity [2].

**Fig. 1:**
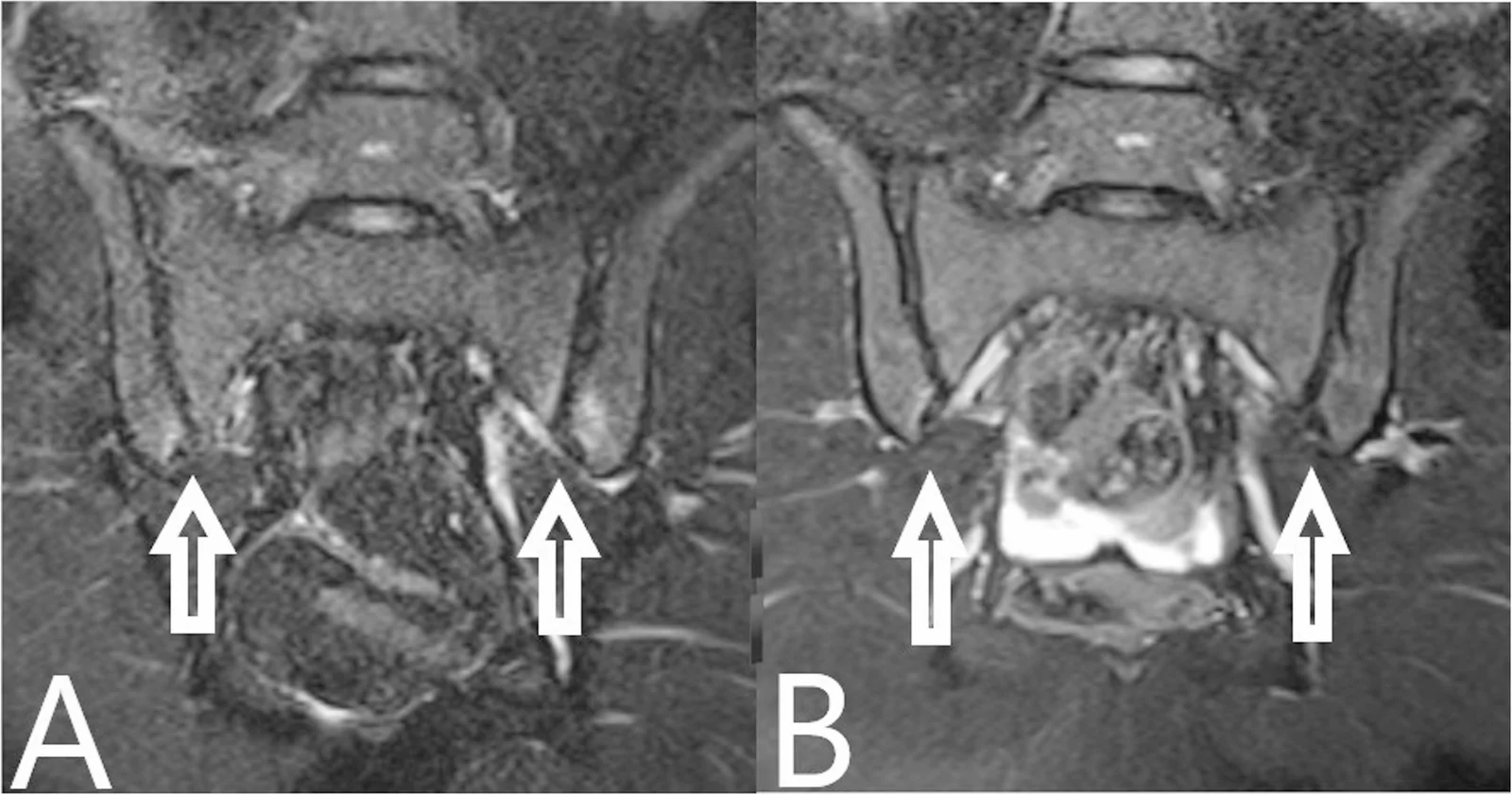
Magnetic resonance imaging (MRI) findings of a patient with isotretinoin-induced sacroiliitis. Bone marrow edema (arrow) observed in Image A has completely resolved in the follow-up scan obtained 3 months later (Image B)

Conversely, isotretinoin, an oral retinoic acid derivative commonly used for the treatment of severe acne vulgaris, has been linked to various musculoskeletal adverse effects. While arthralgia and myalgia are the most frequent manifestations, cases of isotretinoin-induced sacroiliitis have been increasingly reported in the literature [3, 4]. As shown in Fig. 1, the bone marrow edema resolved on the follow-up MRI in the case of isotretinoin-induced sacroiliitis.

In young patients presenting with inflammatory back pain and MRI findings consistent with sacroiliitis, a concurrent history of isotretinoin use poses a considerable diagnostic challenge. Therefore, early and accurate differentiation between these two entities is essential for optimal prognosis, appropriate treatment planning, and avoidance of unnecessary investigations and immunosuppressive therapies. The existing literature mainly consists of case reports describing the imaging characteristics of isotretinoin-associated sacroiliitis, whereas comparative analyses of MRI findings between isotretinoin-induced and axSpA-related sacroiliitis are remarkably limited [5, 6].

The aim of this study was to compare the sacroiliac joint MRI findings of patients with axSpA and those with isotretinoin-induced sacroiliitis and to determine morphological and quantitative MRI parameters that differ significantly between these groups, thereby contributing to differential diagnosis.

## Materials and methods

### Study design and ethical approval

This single-center comparative case–control study was approved by the Clinical Research Ethics Committee of Sanko University Faculty of Medicine (approval date: July 10, 2025; decision no: 2). The study was conducted in accordance with the Declaration of Helsinki.

All consecutive patients who underwent sacroiliac joint MRI at our institution between January 2018 and December 2024 were screened. MRI examinations were retrieved from the PACS archive using the keywords “sacroiliitis”, “sacroiliac joint”, “axial spondyloarthritis” and “isotretinoin”. Corresponding clinical files were reviewed in the hospital information system (HIS). Patients were included sequentially, based on MRI-confirmed sacroiliitis and the fulfillment of group-specific inclusion criteria described below.

### Patient selection

#### Inclusion criteria

- Group 1 (axSpA group; *n* = 40): Patients definitively diagnosed with axSpA in the rheumatology clinic according to the 2009 Modified New York or ASAS classification criteria, who underwent sacroiliac joint MRI for diagnostic or follow-up purposes at our institution. To ensure appropriate comparison with drug-induced cases and to avoid overrepresentation of chronic structural damage, patients with long-standing or advanced axSpA (e.g., established ankylosis or sclerosis) were not included.
- Group 2 (Isotretinoin-associated sacroiliitis group; *n* = 28): Patients receiving oral isotretinoin for acne vulgaris who developed inflammatory low back/buttock pain during treatment or within three months after discontinuation, whose MRI demonstrated active sacroiliitis according to ASAS/OMERACT definitions, and in whom no alternative rheumatic or infectious disease was identified upon rheumatology evaluation. Isotretinoin treatment was administered for 4–6 months at an average daily dose of 30 mg and a total dose of 120–150 mg/kg.

#### Exclusion criteria

• Suboptimal MRI quality precluding evaluation.
• History of trauma, infection (e.g., septic arthritis, brucellosis), or tumor involving the sacroiliac joint.
• Presence of an inflammatory rheumatic disease other than axSpA (e.g., rheumatoid arthritis, SAPHO syndrome).
• History of sacroiliac joint surgery.

### MRI imaging protocol

Sacroiliac joint MRI examinations were performed using a 1.5-T system (Siemens Magnetom Avanto, Siemens Healthineers, Erlangen, Germany). Imaging was acquired with a dedicated protocol optimized for sacroiliac joint assessment, using a small field of view (FOV, 220 mm), 3-mm slice thickness with no interslice gap, and planes oriented parallel to the long axis of the sacrum to ensure optimal visualization of the joint space.

The protocol included coronal oblique T1-weighted turbo spin-echo (TSE) sequences (TR: 400–600 ms, TE: 10–20 ms) for the evaluation of structural lesions such as sclerosis, erosions, and fatty metaplasia; coronal oblique short tau inversion recovery (STIR) sequences (TR: 3500–4500 ms, TE: 30–60 ms, TI: 150 ms) for the detection of active inflammatory changes, particularly bone marrow edema; and axial oblique T2-weighted TSE sequences (TR: 3000–4000 ms, TE: 80–100 ms) for detailed assessment of joint morphology and periarticular structures.

In addition, a large field-of-view coronal T2-weighted sequence (TR: 3000 ms, TE: 90 ms) encompassing the entire pelvis was acquired to identify potential extra-articular or alternative pathologies. This standardized imaging protocol was designed to maximize sensitivity for active inflammatory lesions while allowing comprehensive evaluation of structural changes associated with sacroiliitis.

### Image analysis

All MRI examinations were independently reviewed on a PACS workstation by two musculoskeletal radiologists with 7 and 10 years of experience, blinded to clinical data and diagnostic groups.

MRI evaluation was performed in the coronal oblique plane in accordance with ASAS/OMERACT recommendations. Since patients with long-standing or advanced axSpA were excluded from the study, the assessment focused on active inflammatory lesions and early structural changes.

### Active inflammatory findings

#### Bone marrow edema (BME)

BME was defined according to ASAS/OMERACT criteria as hyperintense signal on STIR and T2 fat-suppressed sequences, detected on at least two consecutive coronal-oblique slices, or involving two distinct subchondral locations within a single slice. This definition was used to avoid misclassification of very small or isolated high-signal areas that may not represent true inflammatory lesions.

For each case, we recorded the presence of bone marrow edema (yes/no), its laterality (right/left/bilateral), its symmetry (symmetric/asymmetric), its predominant surface (iliac/sacral), and its predominant region (superior/inferior).

Extent of BME was determined by measuring the mean width of the largest edema focus on coronal-oblique STIR images and categorizing it as ≥1 cm or <1 cm, consistent with the parameters analyzed in the study.

#### Structural lesions assessed

Because chronic/advanced axSpA cases were deliberately excluded, only early structural changes were evaluated:

- Erosion: Focal cortical bone defects at the articular surface of the sacroiliac joint, characterized by loss of the normal low-signal cortical margin on T1-weighted images, with replacement by adjacent marrow signal, consistent with subchondral bone destruction.
- Fat Metaplasia: Well-demarcated T1W hyperintense lesions ≥ 5 mm.

#### SPARCC scoring

Inflammatory activity of the sacroiliac joints (SIJ) was assessed using the SPARCC SIJ inflammation score, following the methodology recommended in recent studies. The score is calculated on six consecutive coronal-oblique slices through the cartilaginous portion of the SIJ. Each slice is divided into four quadrants, and bone marrow edema (BME) is scored dichotomously in each quadrant (0–1). Additional points are assigned for: Edema depth ≥ 1 cm on each slice (0–1) and increased edema intensity relative to vascular signal (0–1). The total SPARCC score therefore ranges from 0 to 72, with higher values indicating greater active inflammation.

### Statistical analysis

All statistical analyses were performed using SPSS software, version 25.0 (IBM Corp., Armonk, NY, USA). The Shapiro–Wilk test was applied to assess the normality of continuous variables (age and SPARCC score) within each group. Neither variable followed a normal distribution (*p* < 0.05). Therefore, continuous variables were summarized as medians (minimum–maximum), and categorical variables were expressed as counts (n) and percentages (%).

Between-group comparisons were performed using the Mann–Whitney U test for non-normally distributed continuous variables (age and SPARCC score). The Pearson Chi-square test was used to evaluate differences between groups in categorical MRI findings (e.g., presence or absence of lesions, localization). When the expected frequency in any cell was < 5, Fisher’s exact test was applied.

Interobserver agreement for the semi-quantitative SPARCC score was assessed using the intraclass correlation coefficient (ICC). The consistency of all other categorical MRI findings was evaluated using Cohen’s kappa (κ) coefficient. Kappa values were interpreted as follows: <0.40, poor agreement; 0.41–0.60, moderate agreement; 0.61–0.80, substantial agreement; and 0.81–1.00, almost perfect agreement.

Statistical significance was set at *p* < 0.05 for all tests.

## Results

Of the 68 patients included in the study, 40 were assigned to the axSpA group and 28 to the isotretinoin-associated sacroiliitis group. A detailed comparison of demographic characteristics between the groups is provided in Table 1.

**Table 1:** Demographic characteristics of the study groups

Variable	Isotretinoin group (*n* = 28)	axSpA group (*n* = 40)	*p*-value
Age, median (min–max), years	24 (16–44)	30 (20–41)	< 0.001
Sex, male/female, n (%)	5 (17.9) / 23 (82.1)	26 (65.0) / 14 (35.0)	< 0.001

*p*<0.05 was considered statistically significant. axSpA: Axialspondyloarthritis

### Interobserver agreement

In the assessment of interobserver agreement, the intraclass correlation coefficient (ICC) for the SPARCC score indicated substantial agreement (ICC = 0.77). Substantial agreement was observed for structural findings, including erosions (κ = 0.72), fat metaplasia (κ = 0.75), and bone marrow edema larger than 1 cm (κ = 0.91). Interobserver agreement was lower for the assessment of inferior joint involvement (κ = 0.44), representing fair agreement.

### Demographic and quantitative findings

The median age of patients in the axSpA group (30 years) was significantly higher than that in the isotretinoin-associated sacroiliitis group (24 years; *p* < 0.001). A significant difference in sex distribution was observed between the groups: The axSpA group consisted predominantly of men (65%), whereas the drug-induced group consisted mainly of women (82.1%) (*p* < 0.001). The median SPARCC score, representing the quantitative inflammation index, was significantly higher in the axSpA group (median: 23) than in the isotretinoin-associated group (median: 6) (*p* < 0.001).

### Comparative MRI findings

As shown in Table 2, marked differences were observed between the groups regarding structural changes on MRI. Erosions and fat metaplasia were detected in 55% and 75% of patients with axSpA, respectively, whereas none of the patients in the isotretinoin-associated sacroiliitis group exhibited these findings (*p* < 0.001 for both).

**Table 2:** MRI characteristics of sacroiliitis in the study groups

MRI Feature	Isotretinoin group (*n* = 28)	axSpA group (*n* = 40)	*p*-value
Erosion	0 (0%)	22 (55%)	< 0.001 χ²
Fat metaplasia	0 (0%)	30 (75%)	< 0.001 χ²
T1 hypointensity	12 (42.9%)	31 (77.5%)	< 0.004 χ²
Marked T2 hyperintensity	8 (28.6%)	31 (77.5%)	< 0.001 χ²
Edema > 1 cm	7 (25%)	30 (75%)	< 0.001 χ²

Values are presented as number (percentage). χ²: Chi-square test. Marked T2 hyperintensity: Same intensity as the venous plexus

As presented in Table 3, the pattern of sacroiliitis involvement differed significantly between the groups. Bilateral (95%) and symmetric (67.5%) sacroiliac joint involvement were significantly more frequent in the axSpA group compared to the isotretinoin-associated sacroiliitis group (64.3% and 39.3%, respectively; *p* = 0.002 and *p* = 0.021). Involvement of the right sacroiliac joint (100%) and the inferior joint portion (62.5%) was also more common in patients with axSpA than in those with drug-induced sacroiliitis (*p* = 0.03 and *p* = 0.042, respectively).

**Table 3:** MRI patterns of sacroiliitis involvement in the study groups

MRI finding	Isotretinoin group (*n* = 28)	axSpA group (*n* = 40)	*p*-value
SPARCC score, median (min–max)	6 (3–27)	23 (9–62)	< 0.001
Right sacroiliitis, n (%):	22 (78.6%)	40 (100%)	0.03ᶠ
Left sacroiliitis, n (%):	24 (85.7%)	38 (95%)	0.22ᶠ
Bilateral sacroiliitis, n (%):	18 (64.3%)	38 (95%)	0.002
Symmetric involvement, n (%):	11 (39.3%)	27 (67.5%)	0.021χ²
Predominant surface:			
Sacrum	10 (35.7%)	17 (42.5%)	0.574χ²
Ilium	18 (64.3%)	23 (57.5%)	
Predominant region:			
Superior	10 (35.7%)	15 (37.5%)	0.881χ²
Inferior	18 (64.3%)	25 (62.5%)	0.881

Values are presented as median (minimum–maximum) or number (percentage) unless otherwise indicated. ᶠ: Fisher’s exact test; χ²: Chi-square test; SPARCC: Spondyloarthritis Research Consortium of Canada; axSpA: Axial spondyloarthritis

Among other MRI findings, T1 hypointensity (77.5%), T2 hyperintensity (77.5%), and the presence of bone marrow edema greater than 1 cm (75%) were significantly more frequent in the axSpA group compared to the isotretinoin-associated sacroiliitis group (*p* = 0.004, *p* < 0.001, and *p* < 0.001, respectively). No statistically significant differences were observed between the groups in terms of left sacroiliac joint involvement, sacral or iliac predominance, or superior/inferior regional distribution (all *p* > 0.05).

## Discussion

In this study, sacroiliitis associated with axial spondyloarthritis was significantly more severe and more frequently bilateral and symmetric, with predominant involvement of the inferior portion of the sacroiliac joints, compared with isotretinoin-associated sacroiliitis. In addition, structural changes such as erosions and fat metaplasia were observed exclusively in patients with axSpA, representing an important distinguishing feature between the two entities. To our knowledge, available data directly comparing MRI features of isotretinoin-associated sacroiliitis and axial spondyloarthritis are limited; in this context, our study constitutes the largest comparative series reported so far.

**Fig. 2:**
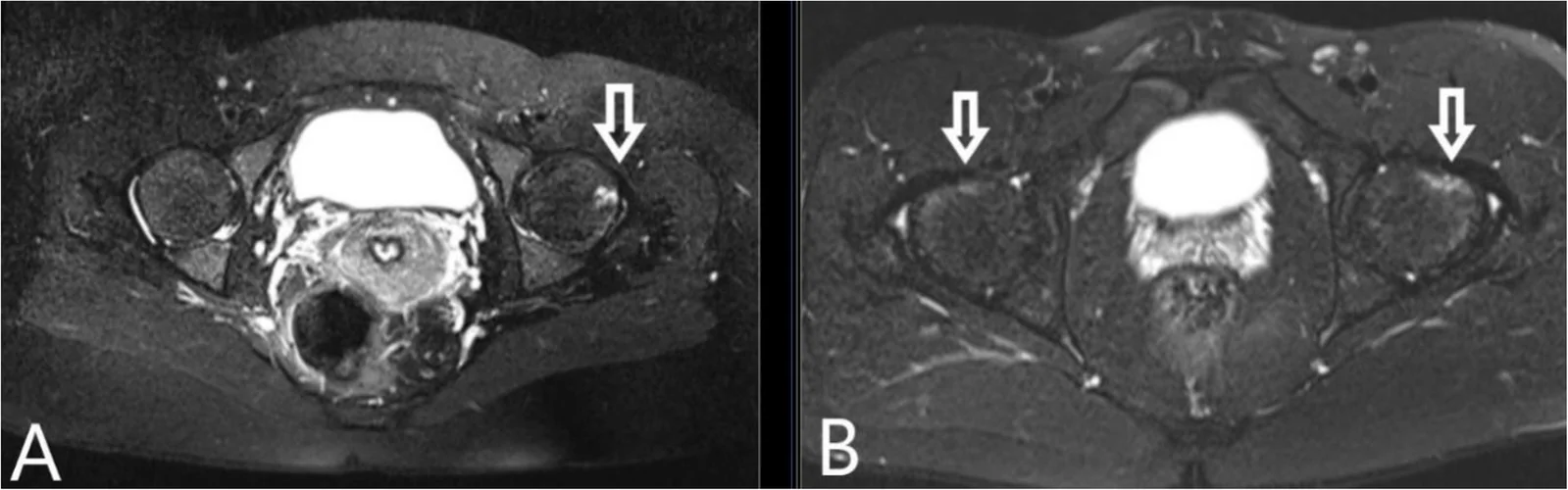
Subchondral bone marrow edema (arrow) at the femoral neck observed in two different cases of isotretinoin-associated sacroiliitis

Although sacroiliitis is a key imaging finding in spondyloarthritis (SpA), it can also be observed in various other conditions encountered in clinical practice, including infections, pregnancy, medication use, and even in healthy individuals [7]. Therefore, distinguishing between these different entities is of great clinical importance. Sacroiliitis associated with axSpA typically demonstrates both active inflammatory and structural features on MRI. Isotretinoin has only rarely been reported to induce sacroiliitis [8]. The isotretinoin-associated form of sacroiliitis is usually bilateral, mild in severity, and tends to resolve completely following discontinuation of the drug (Fig. 1). Isotretinoin may cause arthralgia, arthritis, enthesitis, or sacroiliitis; however, the underlying mechanism remains unclear. It has been suggested that immune dysregulation and disturbances in cytokine balance may contribute to these manifestations. However, Bergler-Czop et al. assessed changes in proinflammatory cytokines during isotretinoin therapy and demonstrated that serum levels of IL-1α, IL-1β, and TNF-α significantly decreased following treatment. These findings suggest that isotretinoin may exert a preventive rather than a predisposing effect on the development of sacroiliitis [9]. However, in another study, Sousa-Canavez and colleagues suggested that isotretinoin-associated cytokine changes were dose-dependent. While cytokine levels decrease at low isotretinoin doses, cytokine levels may probably increase with increasing doses [10]. In our study, all patients remained asymptomatic during the first month of treatment when the isotretinoin dose was low, but symptoms appeared after the dosage was increased. In a prospective study conducted by Alkan et al., SpA-like symptoms associated with isotretinoin therapy were interpreted as paradoxical reactions [11]. In the study by Alkan et al., SpA-like symptoms developed in 23.1% of 42 patients. However, in this series, unilateral sacroiliitis was detected in only 1 case, and enthesopathy was detected in the majority of the other cases. When the anatomical pattern of sacroiliac joint involvement was analyzed, our study confirmed the classical pattern described for axSpA—bilateral, symmetric involvement predominantly affecting the inferior joint segment—thereby supporting the existing literature. However, our findings regarding the regional distribution of involvement revealed a more complex pattern. It is frequently emphasized in the literature that axSPA begins in the inferior region, where enthesitis is dense and biomechanical stress is greatest [12]. In our study, the predominant involvement in the axSPA group was observed in the inferior region, as expected. Interestingly, in the isotretinoin-associated sacroiliitis group, the predominant pattern of involvement was also observed in the inferior region, and no statistically significant difference was found between the two groups in this regard (*p* = 0.881). This finding suggests that the inferior segment of the sacroiliac joint may represent a region inherently more susceptible to inflammation, regardless of the underlying etiology. The finding that contributes most strongly to the differential diagnosis is the presence of structural changes, such as erosions and fat metaplasia, which were detected exclusively in the axSpA group. Although not pathognomonic, this observation indicates a high degree of specificity for the diagnosis of axSpA. These findings likely reflect the processes of endochondral ossification and tissue repair that occur as a result of recurrent inflammatory episodes, which are central to the pathophysiology of axSpA [13]. The complete absence of these chronic markers in the isotretinoin group strongly supports the hypothesis that this entity progresses through a distinct, potentially reversible inflammatory pathway that does not permanently affect bone metabolism, distinguishing it from axSpA. Cases of isotretinoin-associated sacroiliitis reported in the literature are also typically limited to bone marrow edema without evidence of structural damage [5]. A recent study by Erol et al. demonstrated that MRI findings in isotretinoin-associated sacroiliitis regress after discontinuation of the drug, supporting its reversible and non-destructive nature. Our findings are consistent with these observations [14]. The significantly higher median SPARCC score and the presence of bone marrow edema exceeding 1 cm in the axSpA group indicate a more extensive and aggressive inflammatory process compared to isotretinoin-associated sacroiliitis. This finding aligns with the concept that axSpA represents a systemic inflammatory disease, whereas isotretinoin triggers a more localized or reactive inflammatory response. The SPARCC score has been validated as a reliable tool for assessing inflammatory activity and treatment response in axial spondyloarthritis [15]. In light of these findings, a practical diagnostic approach can be suggested for radiologists and clinicians: If only bone marrow edema is present on sacroiliac MRI of a patient taking isotretinoin, the primary diagnosis should be drug-induced sacroiliitis. However, if structural changes such as erosions and/or fat metaplasia accompany the imaging findings, an underlying spondyloarthritis diagnosis should be considered, regardless of isotretinoin use. Interestingly, unilateral femoral neck bone marrow edema was observed in five patients with isotretinoin-associated sacroiliitis (Fig. 2). Although the femoral neck is not routinely included in the standard sacroiliac MRI field of view, this rate was noteworthy.

Transient bone marrow edema associated with isotretinoin use has also been reported in the literature [16]. One of the major strengths of our study is that it is among the first to directly compare these two clinical entities using standardized imaging definitions. The high interobserver agreement for key findings further strengthens the validity of our data. However, the retrospective nature of the study prevents establishing a causal relationship, and its single-center nature limits the generalizability of the results. In addition, the absence of a healthy control group represents another limitation of the study, and future studies including appropriate control groups are needed to better contextualize these findings. Furthermore, sex-related differences in axial spondyloarthritis (axSpA) have been well documented in the literature and may help explain the differences observed between the groups in our study. In addition, the age difference between the groups may also have influenced the observed MRI findings, as increasing age may be associated with longer disease duration and a higher likelihood of structural changes in axial spondyloarthritis. Previous studies have demonstrated that male patients tend to exhibit a higher inflammatory burden and more pronounced structural damage on MRI. In a study by Mayer et al., male patients were found to be significantly more likely to have inflammatory MRI lesions, including bone marrow edema and pelvic enthesitis, compared to females [17]. Similarly, Lorenzin et al. reported that active sacroiliitis was more frequent in male patients on MRI and that males showed greater radiographic progression over time [18]. These findings suggest that male sex is associated with a higher inflammatory load and more aggressive structural involvement in axSpA, which may have contributed to the higher frequency of structural lesions observed in the axSpA group in our study. However, this should also be considered a limitation of our study, as differences in age and gender distribution between the groups may have affected the observed MRI findings. The relatively small sample size and demographic differences between the groups also represent potential limitations. In addition, as the primary focus of this study was radiological comparison, standardized clinical severity scores and laboratory parameters were not systematically assessed in the isotretinoin-associated sacroiliitis group. BASDAI scores were available only for the axSpA group, in which patients with low disease activity (BASDAI < 4) were included. Therefore, clinical comparisons between groups could not be performed.

This study demonstrated that sacroiliitis associated with axSpA and that induced by isotretinoin exhibit distinctly different magnetic resonance imaging characteristics. Findings have the potential to provide valuable guidance to radiologists and clinicians in the differential diagnosis of these two commonly encountered conditions.

## Data Availability

The data supporting this study are available from the corresponding author upon request.
