# Correction to: Comparative radiologic characteristics of isotretinoin-induced sacroiliitis and axial spondyloarthritis

**DOI:** 10.1007/s11845-026-04471-3

**Published:** 2026-06-03

**Authors:** Mehmet Gurdal Demirci, Nurcihan Yavuz Savas, Ozlem Kudas, Muhammed Aslan, Toktamis Savas, Fatma Elif Yildirim, Irada Ibramkhalilova, Fatih Albayrak, Orhan Zengin, Bunyamin Kisacik

**Affiliations:** 1 Department of Radiology, Faculty of Medicine, Sanko University, Gaziantep, Turkey; 2 Department of Rheumatology, Istanbul Physical Medicine and Rehabilitation Training and Research Hospital, Istanbul, Turkey; 3 Department of Internal Medicine, Faculty of Medicine, Sanko University, Degirmicem, Gazimuhtar Pasa Bulvarı No:36, 27090 Sehitkamil/Gaziantep, Turkey; 4 Department of Orthopaedics and Traumatology, Faculty of Medicine, Sanko University, Gaziantep, Turkey; 5 Department of Dermatology, Faculty of Medicine, Sanko University, Gaziantep, Turkey; 6 Division of Rheumatology, Department of Internal Medicine, Faculty of Medicine, Sanko University, Gaziantep, Turkey; 7 Division of Rheumatology, Department of Internal Medicine, Faculty of Medicine, Gaziantep University, Gaziantep, Turkey

**Correction to: Irish Journal of Medical Science (1971 -)**

**https://doi.org/10.1007/s11845-026-04414-y**

The original version of this article was revised.

The names for the two author were incorrectly written;

Incorrect names were Toktamıs Savas and Irada Ibramxalilova

The correct names should read; Toktamis Savas and Irada Ibramkhalilova

The original article has been corrected.

